# Supplementary material for: Splice-Junction-Based Mapping of Alternative Isoforms in the Human Proteome
Source: Cell Rep. Author manuscript; Available in PMC 2020 Jan 15. (PMC6961840; doi:10.1016/j.celrep.2019.11.026)

A

sp|O95425|SVIL_HUMAN|ENSG00000197321|MXE2|867|chr10|29531288|29532172|−1|r420|T1,sp|O95425|SVIL_HUM
RPELCTSHSETPTVDDEEKVDER q value: 2.4551e−05 Tr_novel:TRUE RefSeq_Novel:FALSE
Search result spec prec mz: 546.6526 Actual spec prec mz: 546.65265
Fragments matched per AA: 2.26 Proportion of top 20 peaks matched: 0.45

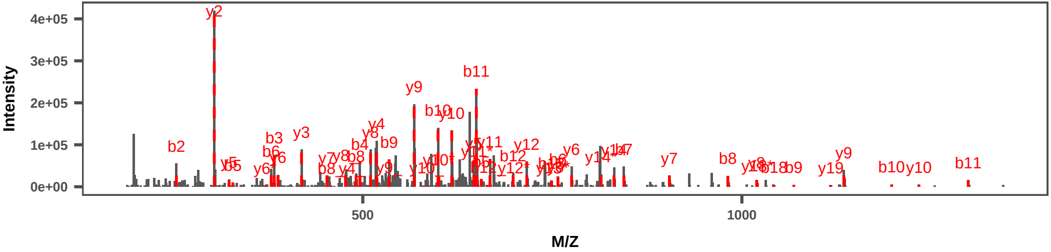

B

Scatterplot of predicted elution time
Fitting R2: 0.848
Novel peptide residual Z score: −0.436
Number of peptides: 1433

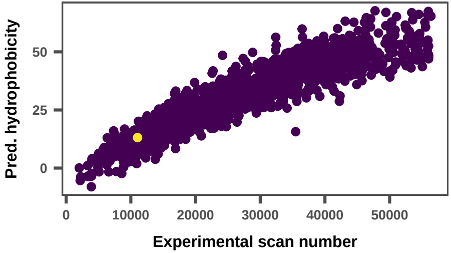

C

Distributions of residuals from best−fit line
of predicted RT vs Expt. scan number
Line: Z score of novel peptide
Z: −0.436

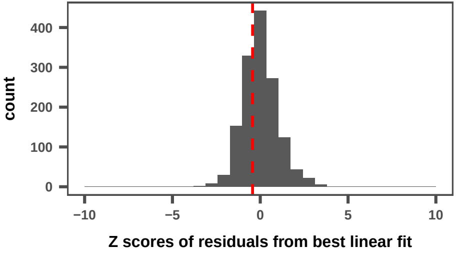

Supplement: 2 [file NIHMS1546469-supplement-2.zip › DF1/PXD010154/Heart/Heart_12_SVIL_RPELCTSHSETPTVDDEEKVDER.pdf]
